# Fission Yeast Sec3 Bridges the Exocyst Complex to the Actin Cytoskeleton

**DOI:** 10.1111/j.1600-0854.2012.01408.x

**Published:** 2012-09-07

**Authors:** Isabelle Jourdain, Hannah C Dooley, Takashi Toda

**Affiliations:** 1 Cell Regulation Laboratory, London Research Institute, Cancer Research UK44 Lincoln's Inn Fields, London, WC2A 3LY, UK

**Keywords:** actin, endocytosis, exocyst, morphology, *Schizosaccharomyces pombe*

## Abstract

The exocyst complex tethers post-Golgi secretory vesicles to the plasma membrane prior to docking and fusion. In this study, we identify Sec3, the missing component of the *Schizosaccharomyces pombe* exocyst complex (*Sp*Sec3). *Sp*Sec3 shares many properties with its orthologs, and its mutants are rescued by human Sec3/EXOC1. Although involved in exocytosis, *Sp*Sec3 does not appear to mark the site of exocyst complex assembly at the plasma membrane. It does, however, mark the sites of actin cytoskeleton recruitment and controls the organization of all three yeast actin structures: the actin cables, endocytic actin patches and actomyosin ring. Specifically, *Sp*Sec3 physically interacts with For3 and *sec3* mutants have no actin cables as a result of a failure to polarize this nucleating formin. *Sp*Sec3 also interacts with actin patch components and *sec3* mutants have depolarized actin patches of reduced endocytic capacity. Finally, the constriction and disassembly of the cytokinetic actomyosin ring is compromised in these *sec3* mutant cells. We propose that a role of *Sp*Sec3 is to spatially couple actin machineries and their independently polarized regulators. As a consequence of its dual role in secretion and actin organization, Sec3 appears as a major co-ordinator of cell morphology in fission yeast.

Two trafficking events, exocytosis and endocytosis, orchestrate the remodeling of the plasma membrane. Exocytosis involves the fusion of intracellular secretory vesicles with the plasma membrane, thereby providing a source of lipid moieties for membrane extension. It is also used to deliver integral membrane proteins at the cell surface and to release material in the extracellular space. Typically, in yeasts, the secretion of hydrolytic enzymes is necessary to dissolve the cross wall (primary septum) between the two daughters cells and complete cytokinesis (1). Most of our current knowledge of polarized secretion comes from studies conducted in the budding yeast *Saccharomyces cerevisiae*. Cargoes emanating from intracellular organelles are transported by motor proteins along cytoskeletal tracks towards polarized areas of the plasma membrane. Sites of vesicle targeting are defined by factors present on the vesicle and by polarity cues at sites of growth (2). The initial contact between the vesicle and the plasma membrane is mediated by tethering factors that are thought to bridge SNAREs (soluble *N*-ethylmaleimide-sensitive factor attachment protein receptor) on apposing membranes. This paired *trans*-SNARE complex docks the vesicle to the receiving membrane and finally induces lipid fusion (3).

The tethering of secretory vesicles before docking and fusion with the plasma membrane is mediated by the exocyst, a conserved octameric protein complex consisting of Sec3, Sec5, Sec6, Sec8, Sec10, Sec15, Exo70 and Exo84 (4–6). The assembly of the exocyst complex is a sequential process. Sec15 is first loaded onto maturing secretory vesicles and may serve to recruit additional exocyst proteins (7,8). Upon delivery of the vesicle at the cell periphery, this pre-complex interacts with membrane-bound Sec3 and Exo70, thus tethering the vesicle to the plasma membrane (9–12).

Endocytosis is responsible for the uptake of extracellular material and the recycling of lipids and surface proteins. Clathrin-mediated endocytosis is a conserved type of endocytosis but contrary to the situation in mammals, membrane invagination in yeasts shows an absolute requirement for actin (13–15). Specialized structures, the actin patches, are dedicated to pulling the forming vesicles inside the cytoplasm (16,17) and are specifically nucleated by the Arp2/3 complex (18,19). In all fungi, actin patches concentrate at sites of active growth and division (20–22). The factors and mechanisms that restrict the localization of actin patches remain largely unknown.

The characteristic rod-like shape of the *Schizosaccharomyces pombe* constitutes an excellent tool by which to study plasma membrane remodeling and cell morphogenesis (23,24). The long, straight axis of fission yeast cells is defined by factors deposited at the cell ends by the microtubule cytoskeleton. In situations where the function or localization of these factors is hindered, cells become curved or develop an ectopic cell tip. However, they remain cylindrical and still grow from their poles (25–28). By contrast, cells lacking Cdc42, a Rho-type GTPase universally involved in cell polarization, lose polarity and become round (29). Cdc42 is believed to be the most upstream polarization cue (24). Some of its effectors are involved in endocytosis, actin nucleation and actin-based transport, and corresponding mutants show a partial loss of polarity (30–33). In a parallel and independent pathway, Cdc42 also controls secretion (34,35) but mutants of the exocyst complex have no shape phenotype (36,37). No ortholog of Sec3 had been so far identified, supporting the fact that the exocyst complex may not control polarity in *S. pombe*. Moreover, *exo70* is not essential for viability and is unlikely to fully compensate the absence of Sec3.

Here we report the identification of the missing *S. pombe* exocyst component Sec3. We first show that fission yeast *Sp*Sec3 plays a canonical role in the tethering of secretory vesicles with the plasma membrane. We then describe an unexpected function of *Sp*Sec3 in actin assembly. We found that via its interaction with actin-binding proteins, *Sp*Sec3 controls the nucleation of actin cables, the localization and internalization of actin patches and the constriction and disassembly of the cytokinetic ring. This dual function of *Sp*Sec3 makes it a key regulator of fission yeast cell morphology.

## Results

We previously identified SPAC17G8.12 open reading frame (ORF) as a new cell-cycle regulator (38). A BLAST search using the product of SPAC17G8.12 against genomes from various species yielded low-probability orthologs of Sec3. For example, the protein only showed 12–13.5% identity with Sec3 from *S. cerevisiae* (12.2%) *Drosophila melanogaster* (13.5%) or *Homo sapiens* (13.2%). We hypothesized that SPAC17G8.12 may share functional features rather than sequence similarities with other Sec3 proteins and designated it *Sp*Sec3 (see below and Figure S1).

### *sec3* temperature sensitive mutants are defective in cell separation

**Figure 1:**
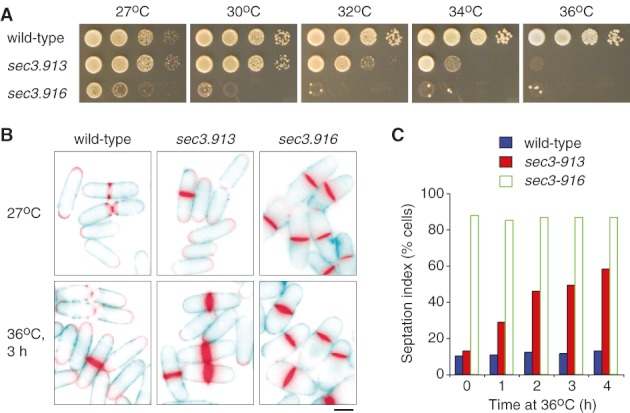
*sec3* temperature sensitive mutants have a septation defect A) Serial dilutions of wild-type, *sec3-913* and *sec3-916* cells were spotted on YE5S plates and incubated at the indicated temperatures. *sec3-916* is more sensitive to high temperatures than *sec3-913*. B) Lectin (blue) and calcofluor (red)-staining of wild-type, *sec3-913* and *sec3-916* cells cultured in parallel at 27 °C and 36 °C. Mutant strains accumulate septated cells at the restrictive temperature and septa appear thick. Bar = 5 µm. C) The percentage of septated cells is shown as a function of incubation time at 36 °C. *sec3-916* (green) has a stronger phenotype than *sec3-913* (red).

As *S. pombe sec3* is an essential gene (39), we created temperature sensitive (ts) alleles by mutagenic polymerase chain reaction (PCR; ts *sec3-913* and *sec3-916*; see *Materials and Methods* for experimental details and Figure S1 for mutation sites). Similar to the situation in *S. cerevisiae*, the ts phenotypes of these mutants were very clear in rich medium but much more attenuated in minimum medium((40)). For this reason and unless stated, most of the experiments presented below were carried out in rich YE5S. *sec3-913* showed wild-type growth and morphology at the permissive temperature 27°C but inhibited growth at higher temperatures (Figure 1A; generation time at 27°C of wild-type = 2 h 40 min and *sec3.913* = 2 h 50 min; at 36°C wild-type = 2 h and *sec3.913* = 13 h 40 min). *sec3-916* exhibited a less conditional phenotype (generation time at 27°C = 7 h 20 min), albeit enhanced at elevated temperatures (no growth at all). No significant drop in cell viability was observed for either mutant within the time frame of the experiments presented in this study.

**Figure 2:**
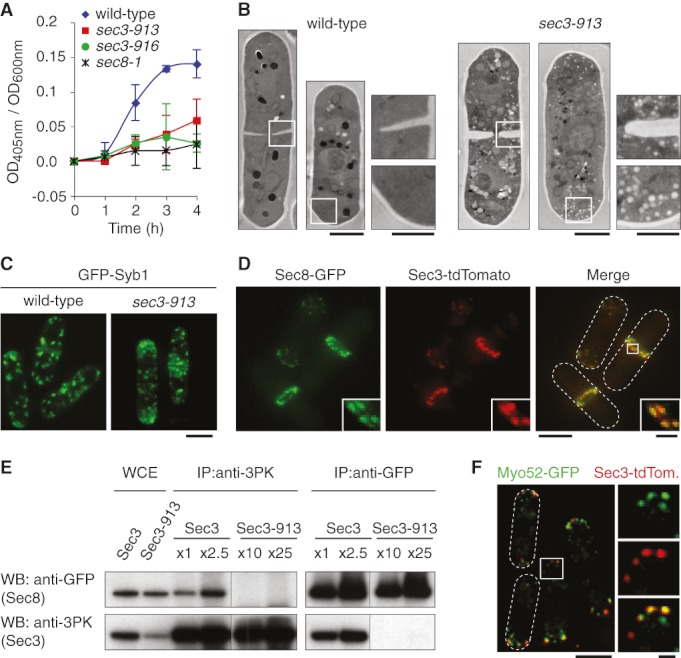
*Schizosaccharomyces pombe* Sec3 is involved in exocytosis A) Activity of acid phosphatase secreted from wild-type (blue diamonds), *sec3-913* (red squares), *sec3-916* (green circles) and *sec8-1* (black crosses) cells. The cumulative activity of the enzyme was measured at time points after the cells were transferred to fresh medium and switched from 27 °C to 36 °C (*t* = 0). Results are shown relative to cell density (OD_600nm_). Error bars indicate the variation between two independent experiments and are SD of the mean. B) EM images of wild-type and *sec3-913* cells. A septated cell and an interphase cell are shown. Note the thick septum in *sec3-913*. Higher magnifications of the boxed areas emphasize the presence of accumulated secretory vesicles in the mutant. Bars = 2 µm. C) Fluorescence microscopy images of the vesicular SNARE Syb1 tagged with GFP and expressed in wild-type and *sec3-913* cells. D) Sec8-GFP and Sec3-tdTomato colocalize at the cell tip(s) and at a medial single or double ring. E) Reciprocal co-immunoprecipitation (IP) between Sec8-GFP and Sec3-3PK or the 3PK-tagged product of *sec3-913*. At equal amounts of whole cell protein extract (WCE) loaded on a gel, the mutant protein was less detectable than wild-type Sec3. For this reason, the amount of protein used in the IP experiment was increased up to 25 times. F) Myo52-GFP and Sec3-tdTomato partially colocalize. Except in panels (D) and (F) for which cells were observed at 27°C, results shown are for cells grown at 36°C. In panels (C, D, F), bars = 5 µm and enlargement bars = 1 µm.

Both mutants had a cell separation defect (Figure 1B and C). In *sec3-913*, the septation index increased gradually over time at 36°C while in *sec3-916* the percentage of septated cells remained constantly high (Figure 1C). By contrast, the septation index of the wild-type strain remained at approximately 11% throughout the experiment. Multiple septated cells were observed in the mutants, with each compartment containing a nucleus (data not shown). Calcofluor-labeled primary septa appeared much thicker in the ts mutants than in the wild-type (Figure 1B). Further analysis of *sec3-913* septa by electron microscopy (EM) showed that septa thickened soon after forming and continued to accumulate cell wall material throughout closure (Figure 2B). Final septum closure and dissolution were also delayed occasionally, leaving an apparent slim cytoplasmic bridge between the two non-separated daughter cells. No cell wall deposition invagination or malformation was otherwise observed at the cell periphery (Figures 1B and 2B).

### *Sp*Sec3 is the missing component of the fission yeast exocyst complex

The septum defects described above could reflect a defect in the delivery and release of hydrolytic septum-specific glucanases (41,42). To test if the secretory pathway was compromised in *sec3* ts mutants, we monitored the activity of acid phosphatase secreted into the medium and reported it relative to cell density (Figure 2A). Similar to the exocyst mutant *sec8-1* (36), the *sec3-916* cells, and to a lesser extent the *sec3-913* cells, secreted less acid phosphatase than the wild-type. A close observation of *sec3-913* cells by EM revealed the cytoplasmic accumulation of vesicles at the cell tips in growing cells and at the cell equator in cells undergoing septation (Figure 2B). Such vesicles were not seen in the wild-type, nor in *sec3-913* at the permissive temperature, presumably due to a higher rate of vesicle fusion with the plasma membrane. We are confident that these structures were post-Golgi secretory vesicles because the v-SNARE Syb1, which normally localizes to scattered, punctate, cytoplasmic structures (Figure 2C, left) (43) was enriched at the cell tips and septum area in *sec3-913* at 36°C (Figure 2C, right). Thus, *Sp*Sec3 is involved in the trafficking of secretory vesicles.

To further characterize Sec3 in fission yeast, we chromosomally tagged it at the C-terminus with fluorescent proteins (GFP or tdTomato) or with 3PK. In a wild-type background at 27°C, *Sp*Sec3 localized to cortical dots capping the growing tip(s) in interphase cells (FiguresD and S2). At cytokinesis, *Sp*Sec3 relocated to the middle of the cell where it first was organized as a single ring. This occurred a few minutes after the contractile actomyosin ring (CAR) was assembled but prior to septin recruitment and primary septum formation (Figure S2). The *Sp*Sec3 ring was not contractile and instead split into two parallel rings at the end of CAR constriction. This localization overlapped perfectly with that of other members of the exocyst complex (Sec8-GFP, Figure 2D and Sec10-GFP, data not shown). To check if *Sp*Sec3 and the exocyst complex physically interacted, we performed reciprocal immunoprecipitations and found that *Sp*Sec3-3PK specifically co-immunoprecipitated with Sec8-GFP (Figure 2E). Genetic data also supported a close functional relationship between *Sp*Sec3 and the exocyst complex, as *sec3-913* was synthetic lethal with *exo70Δ* and with *sec8-1* (Table S2). Hence, *Sp*Sec3 colocalizes and physically and genetically interacts with the exocyst complex.

To verify that *Sp*Sec3 was present at the receiving end of the secretory pathway, we tested whether it colocalized with Myo52, a type V myosin that delivers cargoes from the Golgi apparatus to the plasma membrane (43,44). Indeed, Myo52-GFP and *Sp*Sec3-tdTomato did partially colocalize at the cell cortex (Figure 2F). Overlapping signals presumably illustrate the moment a cargo was delivered by the myosin to the exocyst complex containing *Sp*Sec3, whereas non-coincident signals may reflect the trafficking and recycling of myosin motors toward or from the exocyst complex.

**Figure 3:**
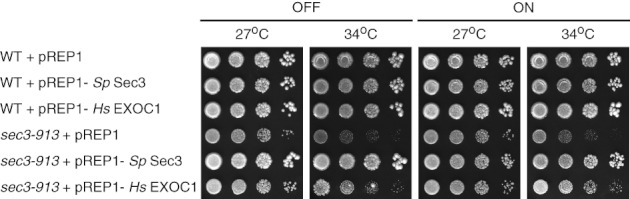
*Schizosaccharomyces pombe* ec3 is the ortholog of *H. sapiens* EXOC1 Serial dilutions of wild-type (WT) and *sec3-913* cells transformed with either an empty plasmid (pREP1), a plasmid containing *S. pombe* Sec3 (pREP1-*Sp*Sec3), or its human ortholog (pREP1-*Hs*EXOC1) were spotted on minimum medium under repressed (OFF, +15 µm thiamine) or expressed conditions (ON, no thiamine). Plates were incubated at the permissive temperature 27°C and at the semi-restrictive temperature 34°C. The temperature sensitivity phenotype of *sec3-913* observed in *sec3-913* + pREP1 is fully rescued by re-expression of *S. pombe* Sec3 and partially rescued by expression of human Sec3/EXOC1.

The above results suggest that *S. pombe* Sec3 is a member of the exocyst complex. During the course of this study, Baek et al. 2010 published the crystal structure of the N-terminal domain of *S. cerevisiae* Sec3. Using this new fold as a template, the authors predicted that *S. pombe* SPAC17G8.12 notably, codes for a Sec3 protein. To unequivocally show that *S. pombe* Sec3 is a functional ortholog of other Sec3 proteins, we asked whether we could substitute *Sp*Sec3 by human Sec3/EXOC1 (Figure 3). Expression of *Sp*Sec3 under the control of the thiamine-repressible *nmt1* promoter rescued the growth phenotype of *sec3-913* at the semi-restrictive temperature 34°C. By contrast, the empty plasmid had no effect. Consolidating our findings with the predictions of Baek's et al. 2010, human EXOC1 expression partially rescued the growth phenotype of the *S. pombe sec3* ts mutant.

Together, our results show that *Sp*Sec3 is the missing component of the fission yeast exocyst complex, which tethers vesicles carried on actin filaments from the Golgi apparatus by a type V myosin to the plasma membrane.

### *S. pombe* Sec3 shares many properties with its orthologs but does not define the site of exocyst assembly

**Figure 4:**
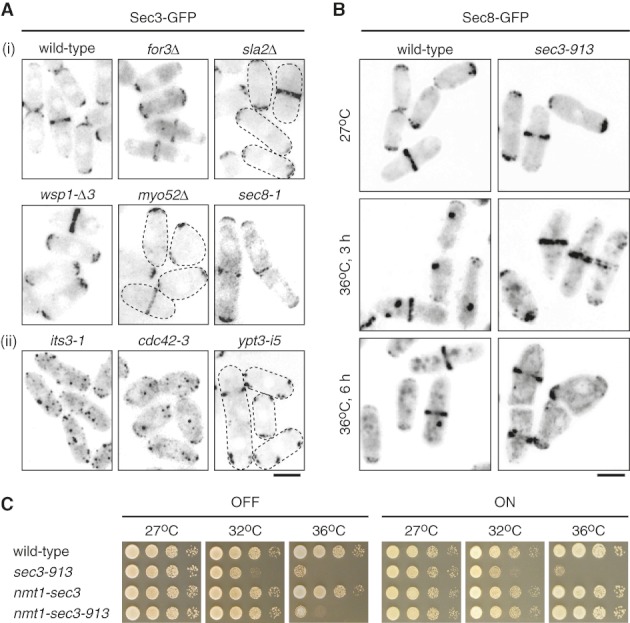
Localization of fission yeast Sec3 A) Sec3-GFP was expressed in the indicated genetic backgrounds. Results for wild-type, *for3Δ*, *sla2Δ*, *wsp1-Δ3*, *sec8-1* and *ypt3-i5*, were obtained at 27°C, at which temperature these strains show a phenotype. *myo52Δ*, *its3-1* and *cdc42-3* were photographed at 36°C. Strains in which Sec3-GFP localization are normal are shown in (i), whereas those displaying delocalized Sec3-GFP are shown in (ii). B) The localization of the exocyst complex does not depend on Sec3. Wild-type and *sec3-913* cells expressing Sec8-GFP were incubated at 27°C or at the restrictive temperature 36°C for up to 6 h. The cytoplasmic aggregates in the wild-type at high temperature have been previously reported (35). Bars = 5 µm, enlargement bar = 1 µm. C) Growth assay showing the phenotype of *sec3-913* is due to its reduced expression. *sec3^+^* and *sec3-913* were over-expressed under the control of the chromosomally integrated, strong *nmt1* promoter (bottom two rows), in repressive (OFF, rich YE5S) or derepressive (ON, minimal medium without thiamine) conditions and at permissive (27 °C) and increasing restrictive temperatures (32–36 °C). Corresponding genes expressed under the control of their own promoter were spotted as controls (top two rows).

Sec3 was proposed to be a landmark for secretion at the bud tip in *S. cerevisiae* (11). In this model, Sec3 localization is independent of the secretory machinery, including vesicle production and transport. To assess if this is also the case in *S. pombe*, we observed *Sp*Sec3-GFP in *for3Δ* cells which lack actin cables (33). We also considered the possibility that actin patches rather than cables may control Sec3 localization and observed it in cells with mis-placed or aberrant patches (*sla2Δ*, *wsp1-Δ3*) (30,46). Finally, we used the *myo52Δ* strain in which vesicular transport along actin filaments is abolished (44) and the *sec8-1* mutant cells, in which vesicle tethering is hindered (36). In all cases *Sp*Sec3-GFP localization appeared identical to that observed in the wild-type (Figure 4A(i)). Hence, in *S. pombe*, as in *S. cerevisiae*, Sec3 localization is not dependent upon either actin or the late secretory machinery.

A crucial aspect of the landmark model is that Sec3 defines the site of exocytosis by targeting other exocyst components to specific sites of the plasma membrane. Challenging this model, Sec8-GFP and other subunits of the exocyst complex were still properly targeted to the tips of *sec3-913* cells incubated at the restrictive temperature for up to two generations (Sec8-GFP, Figure 4B; Sec6-GFP, data not shown). We were unable to detect Sec3-913-GFP at cell tips and middle at the restrictive temperature (data not shown). This could be either due to a technical difficulty to observe Sec3-GFP species at 36°C, the fact that Sec3-913 is delocalized, or the poor stability of the mutant protein. The third was suggested by the fact that *Sp*Sec3-913-3PK was less immunodetected than *Sp*Sec3-3PK in whole cell extracts (WCE, Figures 2E and 7D). Moreover, the re-expression of *Sp*Sec3-913 in the *sec3-913* background using the strong *nmt1* promoter integrated at the endogeneous locus, rescued the ts phenotype of this strain (Figure 4C). Hence, the low levels of Sec3-913 are responsible for the phenotype of *sec3-913* cells. Furthermore, Figure 2E (right panel) shows that *Sp*Sec3-913-3PK is in any case unable to interact with Sec8-GFP. This finding eliminates the possibility that residual levels of mutant Sec3-913 would still be able to recruit Sec8-GFP. We conclude from these experiments that Sec8-GFP localizes properly independently of Sec3. Thus, *S. pombe* Sec3 does not define the site of exocyst assembly.

Sec3 proteins are known to bind directly to the phosphatidylinositol 4,5-bisphosphate (PI(4,5)P_2_) present in the plasma membrane (12,47). PI(4,5)P_2_ is produced by phosphorylation of PI4P, a modification catalyzed in *S. pombe* by the phosphatidylinositol-4-phosphate 5-kinase Its3 (48). In the *its3-1* ts mutant, which has reduced levels of PI(4,5)P_2_, *Sp*Sec3 was displaced from the cell poles and cell equator (Figure 4A(ii)).

The polarization of Sec3 at the *S. cerevisiae* bud tip is mediated by upstream GTPases including Cdc42 (12). Because Cdc42 is also a major orchestrator of morphogenesis in fission yeast (24) we asked whether it could regulate *Sp*Sec3 localization. As expected, Sec3-GFP was detached from the cell tips and middle in the *cdc42-3* mutant (49) (Figure 4A(ii)).

In a search for other factors that may control the polarization of *Sp*Sec3 at cell tips, we identified *Sp*Ypt3. *Sp*Ypt3 is a Rab11-type GTPase involved in endocytic recycling, sorting and transport (50,51). As shown in Figure 4A(ii), *Sp*Sec3-GFP was excluded from the apex of *ypt3-i5* cell tips and aggregated at the vicinity of the plasma membrane. We did not notice any modification of *Sp*Sec3-GFP localization at the equator of these cells.

We conclude from these experiments that *Sp*Sec3 localization and polarization at the plasma membrane are dependent on phopsholipids and on the GTPases Cdc42 and Ypt3. They are, however, independent of secretory vesicle transport, tethering and the actin cytoskeleton.

### *sec3* mutants have a shape phenotype and are hypersensitive to actin poisons

**Figure 5:**
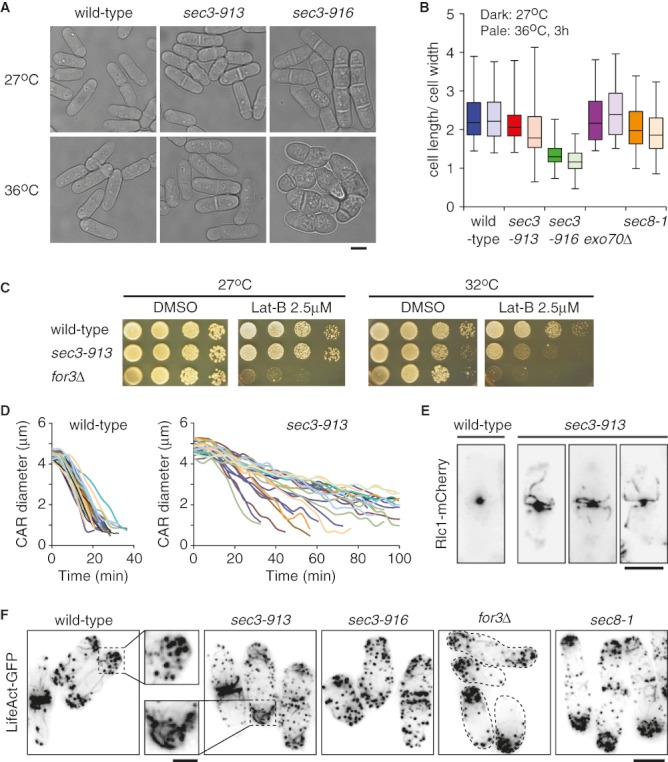
Sec3 controls actin organization and cell morphology in fission yeast A) Phase contrast pictures of wild-type, *sec3-913* and *sec3-916* cells grown at the permissive temperature 27°C, or switched to the restrictive temperature 36°C for one generation. Besides a septation phenotype mutant cells also show an increased diameter. See also Figure 1B. B) Shape phenotype of indicated strains, expressed as the ratio between cell length and cell width. Dark and pale colors show results obtained for cells grown at 27°C or 36°C, respectively. Boxes enclose 50% of the data and lines within the box represent median ratio. Whiskers mark minimum and maximum values. Results are representative of at least two independent experiments. N cells >45. C) Serial dilutions of wild-type, *sec3-913* and *for3Δ* spotted on plates containing either DMSO or 2.5 µm Lat-B and incubated at 27°C or 32°C. The *sec3* mutant is hypersensitive to the actin poison. D) CAR constriction rate measured in wild-type (left panel) and *sec3-913* (right panel) cells grown at 36°C. Results are representative of at least two independent experiments. See also Figure S3. E) Whole cells pictures showing Rlc1-mCherry-containing rings at the end of constriction. Pictures of one wild-type cell and three representative *sec3-913* cells were extracted from time-lapse movies. F) LifeAct-GFP localization in live cells. Actin cables are reduced in *sec3* mutants and patches are delocalized. Cytoplasmic projections of an unclosed ring described in (E) can be seen in the *sec3-913* cytokinetic cell. *sec3-913* cells frequently show short, entangled, actin filaments accumulated at cell tips (higher magnifications of boxed areas). See also Figure S4. Bars = 5 µm and enlargement bar = 2 µm.

Besides a septation phenotype, the *sec3* ts mutants also displayed a shape phenotype (Figure 5A; see also Figure 1B). The diameter of *sec3-913* cells increased after one generation at 36°C and continued to enlarge upon longer incubation. The more severe *sec3-916* mutant displayed the same phenotype even at the permissive temperature. At the restrictive temperature, this phenotype worsened and cells became misshaped. The extent of this enlargement was quantified by measuring the ratio cell length versus cell width (Figure 5B). *sec8-1* cells also showed a shape phenotype, albeit modest, but *exo70Δ* cells did not.

Because this phenotype was reminiscent of an actin defect, we tested the ability of mutant colonies to grow in the presence of the actin antipolymerizing drug Latrunculin-B (Lat-B; (52)). In the growth test in Figure 5C, *for3Δ* cells were used as a positive control. *sec3-913* appears more sensitive to 2.5 µm Lat-B at the semi-restrictive temperature 32°C, than the wild-type. Thus, Sec3 plays a critical role in actin-mediated cell morphogenesis.

In yeasts, F-actin is found in three independent structures, namely the CAR, actin cables and actin patches. We therefore set out to investigate which of these structures could account for the hypersensitivity of *sec3* mutants to the actin poison.

### *Sp*Sec3 orchestrates CAR constriction and disassembly

We first followed actomyosin ring assembly, contraction and disassembly by time-lapse imaging of the myosin light chain Rlc1. In control conditions during mitosis, Rlc1-mCherry organized as a ring from a broad band of nodes, which constricted until the ring was totally closed and looked like a single dot in the middle of the cell. The CAR then disassembled and the Rlc1-mCherry signal vanished. At 36°C, *sec3-913* cells initially formed an apparently normal actin ring but its constriction was substantially delayed (FiguresD and S3). At the end of constriction, the ring failed to close completely, leaving a cytoplasmic bridge between the two daughter cells. Furthermore, Rlc1-mCherry was seen in filamentous extensions that projected from the unclosed ring toward the cytoplasm of each daughter cell (Figure 5E).

### *Sp*Sec3 mediates actin cable organization

To image actin cables, we used the F-actin marker LifeAct-GFP (Figure 5F). In control conditions, actin filaments run along the long axis of the cells. Interestingly, in the *sec3* ts mutants at the restrictive temperature, filaments appeared faint or absent. This result was confirmed by staining of fixed cells with the F-actin probe Bodipy-Phallacidin (Figure S4). Consistent with an absence of actin cables, time-lapse imaging showed that the long linear translocation of Myo52-GFP along actin cables was abrogated in the *sec3* ts alleles (Movie S1). Unlike in *sec3-913*, *sec3-916* and *for3Δ* cells, and as previously reported (35), we could detect actin cables in *sec8-1* cells, but it is possible that those were remnants of the CAR filaments (Figure 5F).

**Figure 6:**
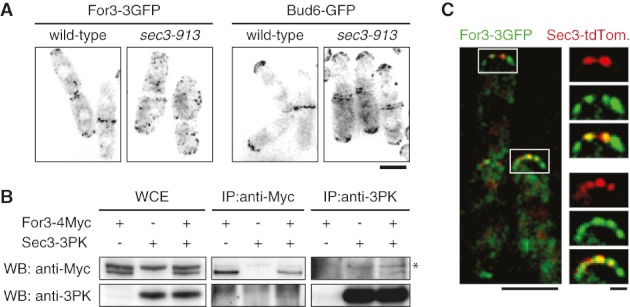
*Sp*Sec3 interacts with the formin For3 and controls its localization A) For3-3GFP, but not Bud6-GFP, is delocalized from the cell tips and cell middle in *sec3-913* cells observed at 36°C. B) Reciprocal co-immunoprecipitation (IP) of Sec3-3PK and For3-4Myc. No interaction was observed with untagged Sec3 or For3. Asterisk: unspecific band. C) Single focal plane picture of cells expressing For3-3GFP and Sec3-tdTomato. Higher magnifications of the boxed areas show that the two proteins partially colocalize. Bars = 5 µm, enlargement bar = 1 µm.

Actin filament nucleation is achieved by the formin For3 (53). Although For3-3GFP was localized at the tips and equator of wild-type cells, in *sec3-913* cells the signal was detached from these regions (Figure 6A). This was not a general consequence of altered cell polarity because the localization of For3-associated and polarisome members Bud6 and Tea1 (54) were not altered in these cells (Figure 6A and data not shown). Reciprocal co-immunoprecipitations further showed that For3-4Myc and Sec3-3PK specifically interact (Figure 6B). This interaction is weak and possibly transient, which may explain why a colocalization between For3-3GFP and Sec3-tdTomato is difficult to visualize *in vivo*. We were nevertheless able to find occasional overlaps between the two proteins (Figure 6C). These results suggest that *Sp*Sec3 docks the formin For3 at the plasma membrane and controls the polarized assembly of actin cables.

Finally, using both LifeAct-GFP (Figure 5F, enlargements) and Bodipy-Phallacidin staining (Figure S4), we noticed an accumulation of entangled actin filaments at the tip of *sec3-913* cells. The nature of these structures and the reason for their aggregation are at present unknown but may reflect defects to release or crosslink newly formed filaments at the cell tip.

### *Sp*Sec3 interacts with the endocytic machinery and controls patch internalization

LifeAct-GFP and Bodipy-Phallacidin also marked the actin patches, which were restricted to the growing tip(s) and to the cell center in control cells. Strikingly, in *sec3-913* at 36°C and in *sec3-916* at any temperature, patches were spread throughout the cell surface (FiguresF and S4).

**Figure 7:**
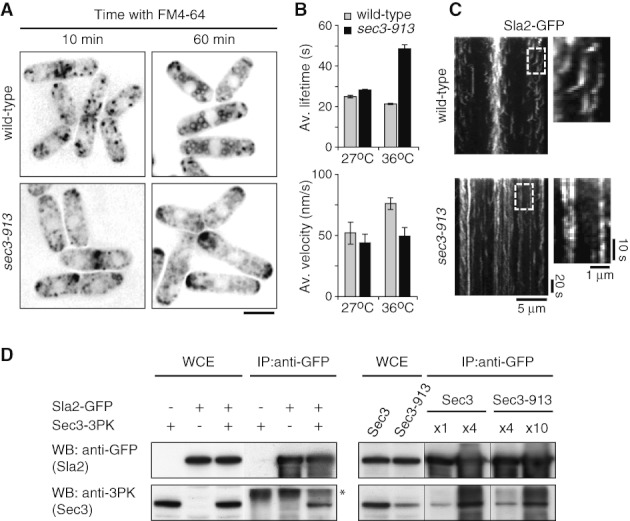
*Sp*Sec3 controls actin patches localization and internalization A) Wild-type and *sec3-913* live cells grown at 36°C were incubated in the presence of FM4-64 and photographed at regular intervals for up to 1 h following addition of the dye. Two selected time points are shown. The accumulation of FM4-64 at the tip of *sec3-913* cells and its absence from endosomes and vacuolar membranes are indicative of a defect in endocytosis. Bar = 5 µm. B) Quantification of the average lifetime (top panel) and average velocity (bottom panel) of Sla2-GFP patches from wild-type (gray) and *sec3-913* (black) cells cultured at the indicated temperatures. N patches >81; N cells >4. Error bars are SEM of individual cells. C) Kymographs of Sla2-GFP signals in representative wild-type (top panel) and *sec3-913* (bottom panel) cells. The *x* and *y* axis represent cell length and time, respectively. Higher magnifications of the boxed areas show that patches are not internalized in *sec3-913* cells. D) Co-immunoprecipitation (IP) of Sec3-3PK with Sla2-GFP. Negative controls include IPs of protein extracts in which either Sec3 or Sla2 was un-tagged (left panel). WCE: whole cell extract. Asterisk: unspecific band. Note that mutant Sec3-913-3PK, although less expressed than wild-type Sec3-3PK, remains capable of interacting with Sla2-GFP (right panel).

Patches in yeasts function in endocytosis and their mislocalization in *sec3* mutants suggested that endocytosis may be affected in these cells. Endocytosis was monitored by following FM4-64 uptake over time (Figure 7A). As described previously (55), upon addition to a culture of wild-type cells, FM4-64 first associates with the plasma membrane at zones of active growth and is incorporated into endosomes which later fuse to the vacuolar membranes. Typically, in wild-type cells the signal at the cell poles decreases rapidly and within 15 min following the addition of FM4-64 <15% of the cells still showed a tip signal. By contrast, FM4-64 incorporation in *sec3-913* endosomes was delayed and virtually all cells still showed tip localization after up to 1 h. Hence, *sec3-913* cells are defective in endocytosis.

To further explore the role of *Sp*Sec3 in endocytosis, we analyzed the dynamic behavior of the actin patch component Sla2 (30) in live cells. At 36°C, patches in *sec3-913* were twice as long-lived as in the wild-type (wild-type = 21.23 ± 4.04 seconds; *sec3-913* = 48.67 ± 6.98 seconds; *n* > 81; Figure 7B). *sec3-913* patches also moved more slowly than wild-type patches (at 36°C, wild-type = 76 ± 5 nm/second; *sec3-913* = 49 ± 7 nm/second; *n* > 81). Similar results were obtained with other actin patch markers (Arc3-mCherry, Chc1-mCherry, Fim1-EGFP, data not shown) (56). To determine which step of endocytosis is defective in the *sec3-913* mutant, we generated kymograph representations of single whole cells over time. In agreement with results presented above (Figure 5F), Sla2-GFP patches were scattered along the long axis of the *sec3-913* cells compared to polar or equatorial localization in wild-type cells (Figure 7C). The kymographic profile of wild-type patches matched previous descriptions, which included the local recruitment of the patch machinery at the cell cortex, the directional internalization of the vesicle away from the cortex and towards the cell interior, and the dissociation of the endocytic machinery from the internalized vesicle events (56). In *sec3-913* cells, the patches were properly assembled at the cell cortex but were not pulled into the cytoplasm nor were they disassembled (Figure 7C). The same conclusions were drawn using Arc3-mCherry (data not shown).

We next asked if the role of *Sp*Sec3 in endocytosis is direct and if it can interact with actin patch components. Figure 7D (left panel) shows that *Sp*Sec3-3PK co-immunoprecipitated with Sla2-GFP. A weak interaction was also detected with Arc3-3HA (data not shown). Although mutant Sec3-913 remained capable of interacting with Sla2 (Figure 7D, right panel), the reduced level of the mutant protein likely explains the dispersion of patches (Figures 2E and 7D).

Taken together these results indicate that Sec3 is involved not only in actin patches localization but also in internalization.

## Discussion

The exocyst is a conserved complex of eight proteins that tethers post-Golgi secretory vesicles to the plasma membrane before docking and fusion. Orthologs of all but one have been characterized or predicted in *S. pombe*. In this study, we identified *Sp*Sec3, the missing component of the fission yeast exocyst complex. We show that *Sp*Sec3 has a role in vesicle tethering that does not involve the recruitment of other exocyst components. *Sp*Sec3 is also required for the organization of all three yeast actin structures, the actin patches, actin cables and CAR. We discuss below how this dual role may be geographically and functionally coordinated and how we envision the role of Sec3 family proteins in membrane trafficking and cell morphogenesis.

### Sec3 is not a landmark for secretion in fission yeast

In the currently accepted mechanism of secretion, Sec3 and Exo70 are present at specialized sites of the plasma membrane where they interact with the six other exocyst subunits associated with the arriving vesicle, thus capturing vesicles and targeting secretion (9–11,47). Our data argue against this simple ‘landmark model’. Indeed, we found that the exocyst subunit Sec8 is polarized independently of its interaction with Sec3. Firstly, Sec8 was properly localized in cells expressing low levels of Sec3-913 mutant protein. Secondly, the ts phenotype of the *sec3-913* strain was rescued by over-expressing the mutant protein, which is otherwise unable to interact with Sec8. This strongly indicates that the secretion defects observed in *sec3* ts mutants are not due to a failure to polarize the exocyst complex. The idea that Sec3 may not target the exocyst to sites of polarized growth is not novel. Similar to our findings, in a budding yeast strain in which the PH domain of Sec3 was deleted, the truncated product was absent from the plasma membrane but the exocyst complex was properly localized (57,58). Furthermore, the polarized recruitment of other exocyst subunits to the *S. cerevisiae* daughter cell is unaffected in a *sec3* strain deleted of its exocyst-binding region (59), and even in a *sec3Δ* strain (60). The exocyst complex may be recruited by Exo70 in the absence of Sec3 (61). However, the possibility of a redundancy between Sec3 and Exo70 is itself disputed (58). The allosteric model proposes that the polarization of the exocyst machinery is achieved by the local activation of a pre-assembled exocyst by regulatory GTPases present at key cortical sites (58). In fact, most, if not all, members of the exocyst complex appear to depend on one another for their localization and/or assembly (6,59,62,63). It is likely that the highly regulated delivery, recruitment and ordered assembly of subunits by one another are responsible for the formation of the whole complex and its targeting to sites of active growth. The role of each subunit and of Sec3 in particular essentially remains to be determined.

### *Sp*Sec3 is required for cell morphology

**Figure 8:**
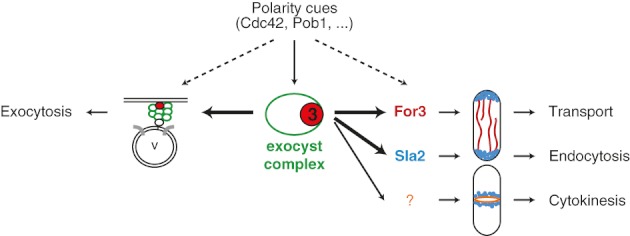
Model for the dual role of *Sp*Sec3 in secretion and actin organization As part of the exocyst complex (green), Sec3 (3, red) has a canonical role in tethering secretory vesicles (v) with the plasma membrane, before docking by SNAREs (gray) and fusion. It also physically binds actin-binding proteins (For3, Sla2 and probably others) and clusters them at the vicinity of their activators (see text). Sec3 therefore bridges the secretion machinery to all three actin structures in fission yeast, the actin cables (red), actin patches (blue) and actomyosin ring (orange). Upstream polarity cues orchestrate the presence of the recruiter Sec3 and the activators in the same areas of the plasma membrane.

The involvement of the exocyst complex in defining a direction of growth and, hence polarity has been established in a range of cell types from budding yeast to growing plant cells and from neurons to cilia (64–66). In fission yeast, cell polarity is not only a simple function of exocytosis but also requires actin cables (35,67,68). In line with this, our *sec3* mutants, which are defective in secretion and possess no actin cables also display a shape phenotype. The current model proposes that the exocyst complex and actin cables are two independent and parallel morphogenesis pathways, both downstream of Pob1 and under the lid of Cdc42 (35,67,68). Our results on the contrary show that the two structures are physically and functionally bridged by Sec3 (Figure 8).

Like *Sp*Sec3, the Boi-family protein Pob1 interacts with both For3 and the exocyst complex (68,69). Pob1 recruits Sec6, Sec8 and Sec10 to cell tips, and our preliminary data suggest that it also positions Sec3. *Sp*Sec3 does not control the recruitment of other exocyst subunits to the plasma membrane. Thus, the function of Pob1 on the exocyst complex is unlikely to be solely through Sec3. Alternatively, Sec3 and Pob1 may play complementary and overlapping roles in polarized growth (68).

The *sec8-1* allele has a much stronger phenotype than any of our two *sec3* mutants (our personal observation), but we and others (35) were able to visualize actin cables in these cells, whose diameter was only mildly enlarged. Similarly, *exo70Δ* cells are not misshapen (35,36). This raises an intriguing question as to whether *Sp*Sec3 is unique among the exocyst complex components. It is formally possible that the lack of other exocyst mutants has precluded a comprehensive analysis of the role of the exocyst as a whole in cell morphology. On the other hand, although the actin patches looked essentially normal in *sec8-1* cells, equatorial but not polar endocytosis was shown to be inhibited in these cells (55). We envisage that *Sp*Sec3 performs multiple tasks by forming various subcomplexes with some, but not all, members of the exocyst complex. Multiple combinations of exocyst components could be a means to provide functional diversity besides secretion, and possibly target different functions to different cellular locations (70).

### Specificity of the actin phenotypes observed in the *sec3* mutants

One of the important findings of this study is that all three fission yeast actin structures were affected in *sec3* ts mutants. The constriction and disassembly of the actomyosin ring were delayed; actin patches were delocalized and failed to invaginate; and actin cables were faint or absent.

The actin phenotype of the *sec3* mutants is not a secondary consequence of their defective secretion. Although not tethered/fused with the plasma membrane, secretory vesicles in the *sec3* mutants accumulated at the cell poles. Thus, if the actin patch and cable components were transported by vesicles, they would also amass in these regions, in a manner similar to Syb1. Instead, we found that they were randomly distributed throughout the cell.

We considered the possibility that *Sp*Sec3 could be responsible for the localization or regulation of some major polarity cues, like Cdc42 or phosphoinositides. However, PI(4,5)P_2_ and their generating enzyme Its3, are not polarized in fission yeast and unlikely to be controlled by Sec3 (48). Moreover, the polarized localization of active Cdc42-CRIB is not affected in *sec3* and *sec8* mutants (data not shown, (35)). Finally, we have shown that, except for actin, a range of other polarity markers (exocyst, v-SNARE, Myosin V, polarisome) were properly localized at zones of growth and division, which would not be the case if Cdc42 was mis-targeted. Hence the role of *Sp*Sec3 in cell morphology is probably not through Cdc42.

Another possibility to explain the ubiquitous effect of Sec3 on actin could be that it specifically controls one structure only, which in turn would indirectly affect the two others. The non-disassembled CAR may sequester a pool of G-actin that would therefore not be available for polymerization into actin filaments or patches (71). This could result from different actin-binding proteins competing for the formation and stabilization of preferential types of actin structures (72,73).

Alternatively, Sec3 may control all three actin structures individually. We favor this hypothesis because *Sp*Sec3 physically interacts both with actin patches components and with the actin cable nucleation machinery. This is in accord with other reports that have shown an association of some members of the exocyst with the endocytic or actin nucleation machineries (74,75). We speculate that Sec3 may also interact with some CAR components, via a mechanism that is common to all three actin structures (see below).

### *Sp*Sec3 couples the formin For3 with its activators

For3 is a typical formin, which contains a Dia inhibitory domain (DID) and a Dia autoregulatory domain (DAD). In a closed conformation, the DID and DAD regions interact with one another and inhibit For3. Upon binding of the DID domain with Pob1 and Cdc42 and of the DAD domain with Bud6, For3 adopts an open and active conformation (69,76). Once de-inhibited, For3 nucleates short actin filaments that will later be bundled into long actin cables (76).

Our results identify *Sp*Sec3 as a new factor which is essential for the localization of For3 at the plasma membrane. The function of For3 is intrinsically linked to its localization and accordingly the *sec3* mutants had no actin cables. For3, but not Bud6 or Cdc42, was delocalized in the *sec3-913* mutant. We therefore conclude that *Sp*Sec3 is at least necessary to tether For3 to the polarized areas of the plasma membrane and to couple it to its activators. It is also possible that Sec3 binds to the DID or the DAD domains and directly relieves For3 autoinhibition in a manner similar to Bud6 and Pob1/Cdc42.

### *Sp*Sec3 is at the meeting point between endocytosis and exocytosis

A role of Sec3 as a link between the cell surface and the actin machinery is also demonstrated by the dispersion of actin patches in the *sec3* mutants.

The delocalization of the patches in the *sec3* mutants is not a consequence of an absence of actin cables. Indeed, patches are hardly delocalized in *for3Δ* cells (33,55,77) and endocytosis is insensitive to concentrations of Latrunculin-A that depolarize cables (55). Moreover, the patches assemble, invaginate and disassemble locally at the plasma membrane (Figure 7C), which supports the idea that patches exist without cables.

Membrane proteins and lipids that have been internalized by endocytosis can be recycled back to the cell surface. A number of studies conducted in various organisms have reported a localization of exocyst subunits in recycling endosomes and their regulation by recycling endosome-specific GTPases such as Arf6 and Rab11 (75,78–81). Although we have not been able to visualize Sec3 in endosomes, its interaction with endocytic patches was detected by immunoprecipitation. Moreover, its localization appeared to be dependent on the Rab11 GTPase Ypt3. It is intuitive that Sec3 drives the tethering of recycling vesicles with the plasma membrane along with the rest of the exocyst complex. How it may control patch internalization is however less clear. It is possible that *sec3* mutants simply fail to recycle factors that define the localization and functionality of actin patches.

All early and late endocytic markers (Chc1, Sla2, Arc3, Fim1 and actin, (56)) that we have tested seemed to assemble into (misplaced) patches. Yet it is clear that these structures were not functional. Their increased lifetime and lower speed in the *sec3* mutants suggest that the patches were waiting for a signal to progress through internalization. The propelling force behind patch invagination is provided by actin. If Sec3 plays a similar role in endocytic patches as it does in actin cables, one can imagine that compromised Sec3 causes the declustering (e.g. by poor recycling) of the assembly-competent actin machinery away from its activator(s). As human Sec3/EXOC1 is able to rescue *Spsec3* mutants, it would be of particular interest to see whether vertebrate Sec3 plays an analogous role in actin organization.

## Materials and Methods

### Yeast genetics and culture

Media, growth, genetics and maintenance of strains were as described in (82). Cells were cultured to mid-log phase in YE5S at 27°C and subsequently shifted to 36°C for 3.5 h before observation unless otherwise stated. For simplicity and unless specified, only experiments carried out at 36°C are shown.

For spot tests all strains were adjusted to the same cell concentration of 2 x 10^7^ cells/mL and 10-fold dilutions were spotted on plates. Plates were incubated at a temperature range of 27–36°C. Lat-B (Calbiochem) was prepared in dimethyl sulphoxide (DMSO) at a concentration of 5 mg/mL (12.64 mm) and diluted into YE5S to 0–10 µm prior to pouring into small 60 × 15 mm dishes. For the rescue experiment of *sec3-913* by human EXOC1, cells transformed with the individual plasmids were grown in minimum medium (nitrogen-based EMM2 or glutamate-based GMM) containing 15 µm thiamine, washed five times in H_2_O and spotted onto EMM or GMM plates with or without 15 µm thiamine. Only selected conditions of temperature, concentration or medium are shown.

### Yeast strains and construction of sec3 temperature sensitive mutants

Yeast strains used in this study are described in Table S1.

For isolation of temperature-sensitive (ts) *sec3* mutants, *sec3* fragments followed by a 3′-Hph^R^ cassette were randomly mutagenized by error-prone PCR amplification using LA-Taq DNA polymerase (Takara). Twenty PCR products (50 μL per tube) were individually ethanol-precipitated and transformed into a wild-type strain. Hph-resistant transformants were then replica-plated on YE5S + phloxin-B plates and incubated at 27°C and 36°C. Eleven clones that grew at 27°C but not at 36°C were selected. These were back-crossed to ensure proper integration and to remove potential off-site mutations. Two alleles, *sec3-913* and *sec3-916*, were chosen for further characterization. Sequencing of *sec3-913* identified two silent mutations (nucleotides A1260G and T1290C), and two missense mutations (nucleotides T448C and A1621C), that resulted in amino acid substitution W150R and T541L. *sec3-916* had two silent mutations (nucleotides T930C and T1671C) and three missense mutations (nucleotides A49C, A50C and T1729C) that lead to two amino acid substitutions (N17P and C577R).

Carboxy-epitope-tagged proteins were generated via chromosomal integration of PCR-amplified fragments (83,84). Some Sec3-tagged fusion proteins rendered cells sick under a number of conditions and were not fully functional. However, as far as we are aware, Sec3-GFP cells grown in rich medium were healthy at all temperatures.

The strain *sec3-913*-3*pk* was generated by transforming a *3pk-kan^R^* PCR fragment into a *sec3-913-hph*^R^ strain. Homologous recombination in 3′ of the ORF was checked by selection in the presence of G418, counter-selection on Hph plates and by colony PCR. The presence of the tag did not modify the ts phenotype (not shown) and the mutant protein migrated at the same size as its wild-type counterpart on denaturing gels. Sec3-913-3PK was however less detectable by immunoblotting than wild type Sec3-3PK (see for example Figure 2E).

Similarly, strain *nmt1-P3-kan^R^-gfp-sec3^+^* and *−sec3-913*, were obtained by transforming an *nmt1-P3-kan^R^-gfp* PCR fragment into a wild-type and *sec3-913-hph*^R^, respectively.

### Plasmid constructs

DNA coding for *S. pombe* Sec3 (SPAC17G8.12; NC_003424.3) was amplified by PCR from wild-type genomic DNA. The cDNA of transcript variant 1 of *H. sapiens* EXOC1 (NP_060731) cloned in pCMV6-XL5 was purchased from Origene and the insert was re-amplified by PCR. In both cases, cloning into pREP1 was achieved by using primers containing *Nde*I and *Bam*HI restriction sites (Sigma-Proligo). All constructs were verified by sequencing.

### Cell imaging and fluorescence microscopy

Live cell imaging was performed in an imaging chamber (CoverWell 20-mm diameter, 0.5-mm deep; Molecular Probes) filled with 800 μL of 2% agarose in YE5S and sealed with a 22 × 22 mm glass coverslip. Cells were imaged using an Olympus IX71 wide-field inverted epifluorescence microscope with the Deltavision-SoftWoRx system (Olympus and Applied Precision Co.), in a temperature controlled environmental chamber. Olympus UPlanSapo 63× or 100× NA 1.4, oil immersion objective were used and images captured with a Coolsnap-HQ digital CCD camera or a Cascade EMCCD 512B camera (Roper Scientific). Pictures of Z-sections were deconvolved and projected. Counts, measurements and image presentations were made using Metamorph (Molecular Devices Corporation) and downloaded to Microsoft Excel or Prism for analysis.

A stock solution of calcofluor white (fluorescent brightener 28 Sigma) was prepared at 5 mg/mL in H_2_O vortexed for up to 24 h and centrifuged to eliminate the undissolved powder. One microliter of this suspension was pipetted onto cells pre-applied to an agarose pad, and a coverslip was sealed. Cells were observed immediately.

Where two strains were visualized simultaneously on the same microscope slide, identification of the wild-type was achieved by pre-staining with fluorescent lectin. Soybean lectin AlexaFluor 594 or 488 conjugate (Molecular Probes) was dissolved at 2 mg/mL in H_2_O. One microliter of this stock solution was added to 500 μL cells (to a final concentration of 4 ng/μL). After 10–30 min incubation in the dark, cells were washed at least five times with YE5S, mixed with the unstained strain and observed immediately.

For live-cell imaging of actin patches speed was favored and three optical sections only, spanning a total of 0.6 µm below the plasma membrane were acquired every 0.5 seconds (Arc3-mCherry) or 1–2 seconds (Sla2-GFP).

Rlc1-mCherry expressing cells were incubated at 27°C or at 36°C for 2 h prior and imaged for another 2 h at the respective temperature. Z-stacks were acquired every 4 min.

Endocytosis was monitored by following FM4-64 uptake over time using a protocol described previously (55,85) with minor modifications. In brief, FM4-64 (Molecular Probes) was dissolved in DMSO at a concentration of 1.64 mm (1 mg/mL). At time *t* = 0, 2 μL of this stock solution were added to 400 μL cells (to a final concentration of 8.2 µm). The cells were quickly applied to the agarose pad and photographed at various time points. The efficiency of endocytosis was evaluated by scoring the number of cells at each time point that had taken up FM4-64 and delivered it to vacuoles.

For staining of the actin cytoskeleton, cells were fixed for 1 h in 3% paraformaldehyde. After three washes in PEM buffer (200 mm Pipes, 2 mm EGTA, 2 mm MgSO_4,_ pH 6.9) the cell wall was digested for 20 min at 37°C with 500 μL of 3 mg/mL zymolyase-20T re-suspended in PEMS (PEM + 1.2 m d-Sorbitol). Cells were permeabilized with 500 μL of 1% Triton X-100 in PEMS, washed thrice in PEMS and were incubated for 30 min with constant mixing in a solution containing 50 μL PEM and 1 μL BODIPY-FL-Phallacidin 6.6 µm (200 U/mL dissolved in DMSO).

### Acid phosphatase secretion assay

Acid phosphatase secretion was assayed as described previously (36,86,87) with minor modifications. In brief, cells were grown to mid-log phase in YE5S at 27°C and all strains were adjusted to the same concentration. Cultures were washed twice in medium and split into two halves for further incubation at 27°C and 36°C. At indicated time points, the absorbance of the culture was measured at 600 nm. In parallel, a 500 μL sample was incubated for 12 min at 30°C in the presence of 500 μL substrate solution (2 mm *p*-nitrophenyl phosphate 0.1 m sodium acetate pH 4.0 pre-warmed to 30°C). The reaction was stopped by addition of 500 μL of 1 m NaOH. OD_405nm_ was measured at each time point, using a sample with no cell as a blank. Values of acid phosphatase activity were expressed as a function of cell density and all curves were adjusted to the same value at *t* = 0.

### Co-immunoprecipitation

Cells were lysed in extraction buffer [50 mm HEPES, 50 mm NaF, 50 mm Na-β-glycerophosphate, 5 mm EGTA, 5 mm ethylenediaminetetraacetic acid (EDTA), 0.2% Triton X100, 1× Protease Inhibitor Cocktail, 1 mm phenylmethylsulphonyl fluoride (PMSF)] by the mechanical action of acid-washed glass beads [FastPrep FP120 apparatus (Savant. Co.) 2× 25 seconds, power 5.5]. Debris were eliminated by centrifugation for 1 min, then 5 min at 13 000× *g* at 4°C. Protein concentrations were determined by Bradford assay (Biorad). For co-immunoprecipitations, and unless stated otherwise, 4–10 mg of WCE was incubated for 2 h at 4°C with protein-A Dynabeads coated with antibodies against 3PK (mouse monoclonal Serotec), Myc (rabbit polyclonal, Babco) or GFP (rabbit polyclonal, Invitrogen). Beads were then extensively washed (50 mm Tris–HCl, pH = 7.4, 1 mm EDTA, 150 mm NaCl, 0.05% NP-40, 10% Glycerol, 1 mm DTT, 1.5 mm PNPP, 1× Protease Inhibitor Cocktail, 0.1 mm PMSF) and boiled for 5 min in Laemmli sample buffer. Thirty micrograms of WCE was similarly treated and used as controls. Proteins were separated on denaturing 4–12% gradient gels (BioRad) transferred onto PVDF membranes which were further blocked and immunoblotted in the presence of 10% nonfat milk. Primary antibodies against 3PK (mouse monoclonal, Serotec), Myc (9E10 mouse monoclonal, Babco) or GFP (mouse monoclonal, Roche) were used diluted to 1:1000 in ImmunoShot Solution 1 (2B Scientific). For secondary antibodies, anti-mouse horseradish peroxidase (HRP)-conjugated (GE-Healthcare) or TrueBlot (eBioscience) were used at a dilution of 1:2000–1:4000 in ImmunoShot Solution 2 (2B Scientific). Signals were detected using enhanced chemiluminescence (ECL; GE Healthcare).

### Electron microscopy

Samples were cryofixed by high-pressure freezing using a Leica EM PACT2. Freeze substitution was performed in anhydrous acetone containing 0.01% osmium tetraoxide, 0.1% glutaraldehyde and 0.25% uranyl acetate for 3 days at −90°C using a Leica EM AFS. Freeze-substituted cells were gradually warmed to −20°C and were finally infiltrated with Epon. Serial thin sections of ∼60 nm were cut using a Leica Ultracut UCT microtome and placed on pioloform-coated slot grids. Sections were post-stained with 2% uranyl acetate in 70% methanol for 4 min and with lead citrate for 1 min. Images were acquired with a FEI Tecnai Biotwin electron microscope and Gatan DigitalMicrograph. The captured images were processed with Adobe Photoshop CS2 (version 9.0).

## Supporting Information

**Table S1:** *Schizosaccharomyces pombe* strains used in this study

**Table S2:** Genetic interactions between *sec3* and other genes

**Figure S1: Partial alignment of the primary sequences of *S. pombe* and *S. cerevisiae* Sec3.** *S. pombe* Sec3 (*Sp*, 603 aa) is approximately twice as short as its budding yeast ortholog (*Sc*, 1136 aa). Only regions of special interest are shown. Arrows limit the PH domain (aa 71–241 in *Sc*; (45)). Crystallized (*Sc*) or predicted (*Sp*) secondary structures are boxes in pink (α-helices) and blue (β-strands; (45)). Experimentally characterized amino acids responsible for Rho1-binding are shown in green (88). The three clusters of phosphoinositides-interacting residues are shown in red (88). The Cdc42-binding region is colored in orange (12). Mutations in the fission yeast *sec3* mutants used in this study are highlighted in cyan (*sec3-913*) and in magenta (*sec3-916*).

**Figure S2: Localization of Sec3 during the fission yeast cell cycle.** Time-lapse of Sec3-tdTomato imaged together with the CAR marker Myo2-GFP (A) the septin Spn3-GFP (B) or the calcofluor-stained cell wall (C). For each marker three different panels show three different cells at progressive stages of mitosis and cytokinesis. Sec3-tdTomato is localized at growing tips in interphase (arrows) and as a single (open arrowhead), then double (plain arrowheads) medial ring at cytokinesis. Time indicated in minutes. Bars = 5 µm.

**Figure S3:** *SpSec3* controls CAR constriction

Time-lapse imaging of the CAR marker Rlc1-mCherry observed in representative wild-type and *sec3-913* cells, at 27°C or 36°C. Four minutes time increments are shown. Arrowheads point at projections of CAR filaments into the cytoplasm, also shown in Figure 5E. Bar=5 μm.

****Figure S4:** Unlike *sec8-1, sec3* ts mutants have weak actin cables and delocalized actin patches.** Bodipy-Phallacidin staining of fixed wild-type, *sec3-913, sec3-916 and sec8-1* cells grown at 27°C or at 36°C for 3.5 or 6 h. See also Figure 5F. Bar = 5 μm.

**Movie S1:** Time-lapse imaging of Myo52-GFP in wild-type (left) and *sec3-913* (right) cells grown at 36 °C for 3.5 h. Arrowheads point at examples of moving motors along actin cables. Note the near absence of long-range movement in *sec3-913* cells. Time indicated in sec:min. Bar = 5 µm.
