# Supplementary material for: Fission Yeast Sec3 Bridges the Exocyst Complex to the Actin Cytoskeleton
Source: Traffic. 2012 Sep 7;13(11):1481–95. doi: 10.1111/j.1600-0854.2012.01408.x (PMC3531892; doi:10.1111/j.1600-0854.2012.01408.x)
Supplement: Supplementary file 1 [file tra0013-1481-SD5.doc]

**Supplementary Table 1: *S. pombe* strains used in this study**

a : auxotrophy

h : mating type

ND : not determined

| **Strains** | **Genotype** | **Source** |
| --- | --- | --- |
|  |  |  |
| IJ137 | *ade6.M216 leu1.32 ura4-D18* h- | Lab stock |
| IJ767 | *sec3-913-hph*^R^ *ade6.M216 leu1.32 ura4-D18* h- | This study |
| IJ1032 | *sec3-916-hph*^R^ *ade6.M216 leu1.32 ura4-D18* h- | This study |
| IJ612 | *sec3+-gfp-kan*^R^*ade6.M216 leu1.32 ura4-D18* h+ | This study |
| IJ660 | *sec3+-tdTomato*-*hph*^R^ *leu1.32* h- | This study |
| IJ771 | *sec3+-3pk*-*kan*^R^ *ade6.M216 leu1.32 ura4-D18* h+ | This study |
| IJ1025 | *sec3-913-3PK-kan*^R^ *ade6.M216 leu1.32 ura4-D18* h+ | This study |
| IJ1143 | *nmt1-P3-kan*^R^*-gfp-sec3+ ade6.M216 leu1.32 ura4-D18* h- | This study |
| IJ1144 | *nmt1-P3-kan*^R^*-gfp-sec3-913-hphR ade6.M216 leu1.32 ura4-D18* h- | This study |
| IJ869 | *sec8+-gfp*-*ura4+ leu1-32* h- | Lab stock |
| IJ713 | *sec8+-gfp*-*ura4+* *sec3+-tdTomato*-*hph*^R^ *leu1.32* h=ND | This study |
| IJ724 | *sec8+-gfp*-*ura4+ sec3-913-hph*^R^ a=ND h=ND | This study |
| IJ990 | *sec8+-gfp*-*ura4+* *sec3+-3pk*-*kan*^R^ a=ND h=ND | This study |
| IJ1046 | *sec8+-gfp*-*ura4+ sec3-913-3pk-kan*^R^ a=ND h=ND | This study |
| IJ253 | *gfp-syb1+-kan*^R^ a=ND h- | Lab stock |
| IJ1069 | *gfp-syb1+*-*kan*^R^ *sec3-913-hph*^R^ a=ND h=ND | This study |
| IJ86 | *sla2+-gfp*-*kan*^R^ *ade6-M210 leu1-32 ura4-D18* h- | Lab stock |
| IJ799 | *sla2+-gfp*-*kan*^R^ *sec3-913-hph*^R^ *ura4-d18 leu1-32 ade6-M21?* h=ND | This study |
| IJ1019 | *sla2+-gfp*-*kan*^R^ *sec3-916-hph*^R^ a=ND h=ND | This study |
| IJ1100 | *sla2+-gfp*-*kan*^R^ *sec3+-3pk*-*kan*^R^ *ade6- leu1-32 ura4-D18* h=ND | This study |
| IJ1150 | *sla2+-gfp*-*kan*^R^ *sec3-913-3pk-kan*^R^ *ade6- leu1-32 ura4-D18* h=ND | This study |
| IJ14 | *myo52+-gfp-kan*^R^ *ade6.210 leu1.32 ura4.D18* h- | Lab stock |
| IJ1071 | *myo52+-gfp-kanR sec3-913-hphR ade6.21? leu1.32 ura4.D18* h=ND | This study |
| YSM972 | *for3+-3gfp-kan*^R^ *ade6.216 leu1.32 ura4.D18* h- | S. Martin |
| IJ1154 | *for3+-3gfp-kan*^R^ *sec3-913-hph*^R^ *ade6- leu1.32 ura4.D18* h=ND | This study |
| YSM1057 | *for3+-4myc-kan*^R^ *ade6- leu1.32 ura4.D18* h+ | S. Martin |
| IJ1155 | *for3+-4myc-kan*^R^ *sec3-913-3pk-kan*^R^ *ade6- leu1.32 ura4.D18* h=ND | This study |
| PN4374 | *bud6+-gfp-kan*^R^ *leu1.32 ura4.D18 h-* | P. Nurse |
| IJ909 | *bud6+-gfp-kan*^R^ *sec3-913-hph*^R^ *ade? leu1.32 ura4.D18* h=ND | This study |
| MBY6663 | *pAct1-Lifeact-gfp::leu+* h+ | M. Balasubramanian |
| IJ1122 | *pAct1-Lifeact-gfp::leu+ sec3-913-hph*^R^ a=ND h=ND | This study |
| IJ1124 | *pAct1-Lifeact-gfp::leu+* *sec3-916-hph*^R^ a=ND h=ND | This study |
| IJ1134 | *pAct1-Lifeact-gfp::leu+ sec8-1* a=ND h=ND | This study |
| IJ1135 | *pAct1-Lifeact-gfp::leu+ for3::kan*^R^ a=ND h=ND | This study |
| SO3516 | *rlc1+-mCherry*-*ura4+ ade6- leu1-32 ura4-D18* h- | S. Oliferenko |
| IJ1029 | *rlc1+-mCherry*-*ura4+ sec3-913-hph*^R^ *ade6.M210 leu1.32 ura4.D18* h=ND | This study |
| MBY887 | *sec8-1 leu1-32 ura4-D18* h+ | M. Balasubramanian |
| IJ896 | *sec8-1* *sec3+-gfp-kan*^R^ *leu1-32 ura4-D18* h=ND | This study |
| IJ10 | *myo52::ura4+ ade6.210 leu1.32 ura4.D18* h- | Lab stock |
| IJ613 | *myo52::ura4+ sec3+-gfp-kan*^R^ *ade6.210 leu1.32 ura4.D18* h- | This study |
| IJ83 | *sla2::kan*^R^ *ade6-M210 leu1-32 ura4-D18* h- | Lab stock |
| IJ1052 | *sla2::kan*^R^ *sec3+-gfp-kanR ade6- leu1-32 ura4-D18* h=ND | This study |
| IJ107 | *wsp1::ura4+ leu1-32 ade6-210 ura4-* h+ | Lab stock |
| IJ1049 | *wsp1::ura4+* *sec3+-gfp-kan*^R^ a=ND h=ND | This study |
| IJ47 | *for3::kan*^R^ *ade6-M210 leu1.32 ura4-D18* h+ | Lab stock |
| IJ899 | *for3::kan*^R^ *sec3+-gfp-kan*^R^ *ade6- leu1.32 ura4-D18* h=ND | This study |
| CA5041 | *cdc42.3-kan*^R^ + pREP1-*cdc42 leu1.32 ura4-D18* h- | K. Shiozaki |
| IJ911 | *cdc42.3-kan*^R^ *sec3+-gfp-kanR* a=ND h=ND | This study |
| KP692 | *its3-1 leu1.32 ura4-D18* h- | T. Kuno |
| IJ895 | *its3-1* *sec3+-gfp-kan*^R^ a=ND h=ND | This study |
| IJ904 | *ypt3-i5 leu1.32* h- | Lab stock |
| IJ918 | *ypt3-i5* *sec3+-gfp-kan*^R^ a=ND h=ND | This study |
