# Supplementary material for: Fission Yeast Sec3 Bridges the Exocyst Complex to the Actin Cytoskeleton
Source: Traffic. 2012 Sep 7;13(11):1481–95. doi: 10.1111/j.1600-0854.2012.01408.x (PMC3531892; doi:10.1111/j.1600-0854.2012.01408.x)
Supplement: Supplementary file 2 [file tra0013-1481-SD6.doc]

**Supplementary Table 2: genetic interactions between *sec3* and other genes**

N/A : not applicable

blank : not determined

SL : synthetic lethal

SS : synthetic sick

WT : no genetic interaction

^1^ : this study

^2^ :

^3^ :

|  | ***sec3-913*** | ***sec8-1*** | ***exo70Δ*** | ***for3Δ*** | ***sla2Δ*** | ***cdc42.3*** |
| --- | --- | --- | --- | --- | --- | --- |
| ***sec3-913*** | N/A | SL^1^ | SL^1^ | SL^1^ | SL^1^ | SS^1^ |
| ***sec8-1*** | SL^1^ | N/A | SL^1,2^ | SL^2^ |  |  |
| ***exo70Δ*** | SL^1^ | SL^1, 2^ | N/A | SL^2^ |  |  |
| ***for3Δ*** | SL^1^ | SL^2^ | SL^2^ | N/A |  |  |
| ***sla2Δ*** | SL^1^ |  |  |  | N/A |  |
| ***cdc42.3*** | SS^1^ |  |  |  |  | N/A |
| ***tea1Δ*** | WT |  |  | SS^3^ |  |  |
