# Supplementary material for: Fission Yeast Sec3 Bridges the Exocyst Complex to the Actin Cytoskeleton
Source: Traffic. 2012 Sep 7;13(11):1481–95. doi: 10.1111/j.1600-0854.2012.01408.x (PMC3531892; doi:10.1111/j.1600-0854.2012.01408.x)
Supplement: Supplementary file 3 [file tra0013-1481-SD1.doc]

**Supplementary Figures**

**
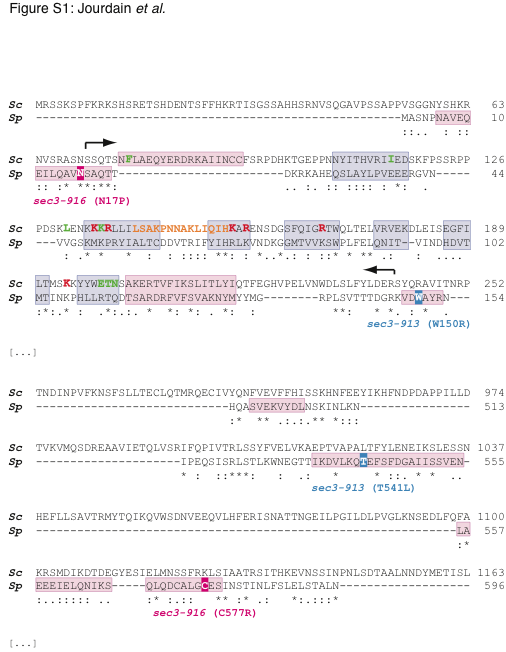
**

**Figure S1: Partial alignment of the primary sequences of *S. pombe* and *S. cerevisiae* Sec3.**

*S. pombe* Sec3 (*Sp*, 603 aa) is approximately twice as short as its budding yeast ortholog (*Sc*, 1136 aa). Only regions of special interest are shown. Arrows limit the PH domain (aa 71-241 in *Sc*; ). Crystallized (*Sc*) or predicted (*Sp*) secondary structures are boxes in pink (α-helices) and blue (β-strands) . Experimentally characterized amino acids responsible for Rho1-binding are shown in green . The three clusters of phosphoinositides-interacting residues are shown in red . The Cdc42-binding region is colored in orange . Mutations in the fission yeast *sec3* mutants used in this study are highlighted in cyan (*sec3-913*) and in magenta (*sec3-916*).
