# Supplementary material for: Fission Yeast Sec3 Bridges the Exocyst Complex to the Actin Cytoskeleton
Source: Traffic. 2012 Sep 7;13(11):1481–95. doi: 10.1111/j.1600-0854.2012.01408.x (PMC3531892; doi:10.1111/j.1600-0854.2012.01408.x)
Supplement: Supplementary file 4 [file tra0013-1481-SD2.doc]

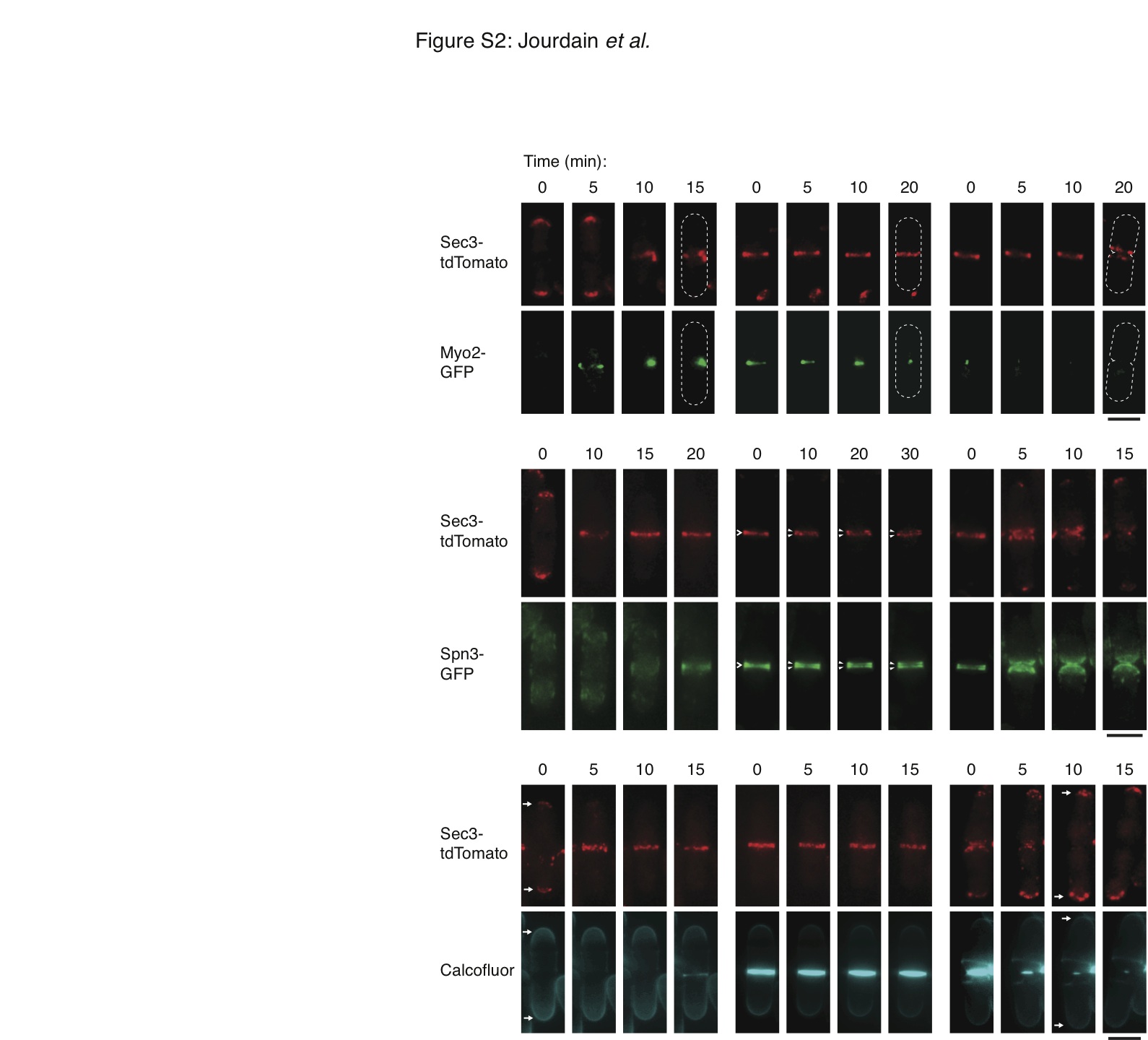


**Figure S2: Localization of Sec3 during the fission yeast cell cycle**

Time-lapse of Sec3-tdTomato imaged together with the CAR marker Myo2-GFP (A) the septin Spn3-GFP (B) or the calcofluor-stained cell wall (C). For each marker three different panels show three different cells at progressive stages of mitosis and cytokinesis. Sec3-tdTomato is localized at growing tips in interphase (arrows) and as a single (open arrowhead), then double (plain arrowheads) medial ring at cytokinesis. Time indicated in minutes. Bars = 5 μm.
