# Supplementary material for: Fission Yeast Sec3 Bridges the Exocyst Complex to the Actin Cytoskeleton
Source: Traffic. 2012 Sep 7;13(11):1481–95. doi: 10.1111/j.1600-0854.2012.01408.x (PMC3531892; doi:10.1111/j.1600-0854.2012.01408.x)
Supplement: Supplementary file 5 [file tra0013-1481-SD3.doc]

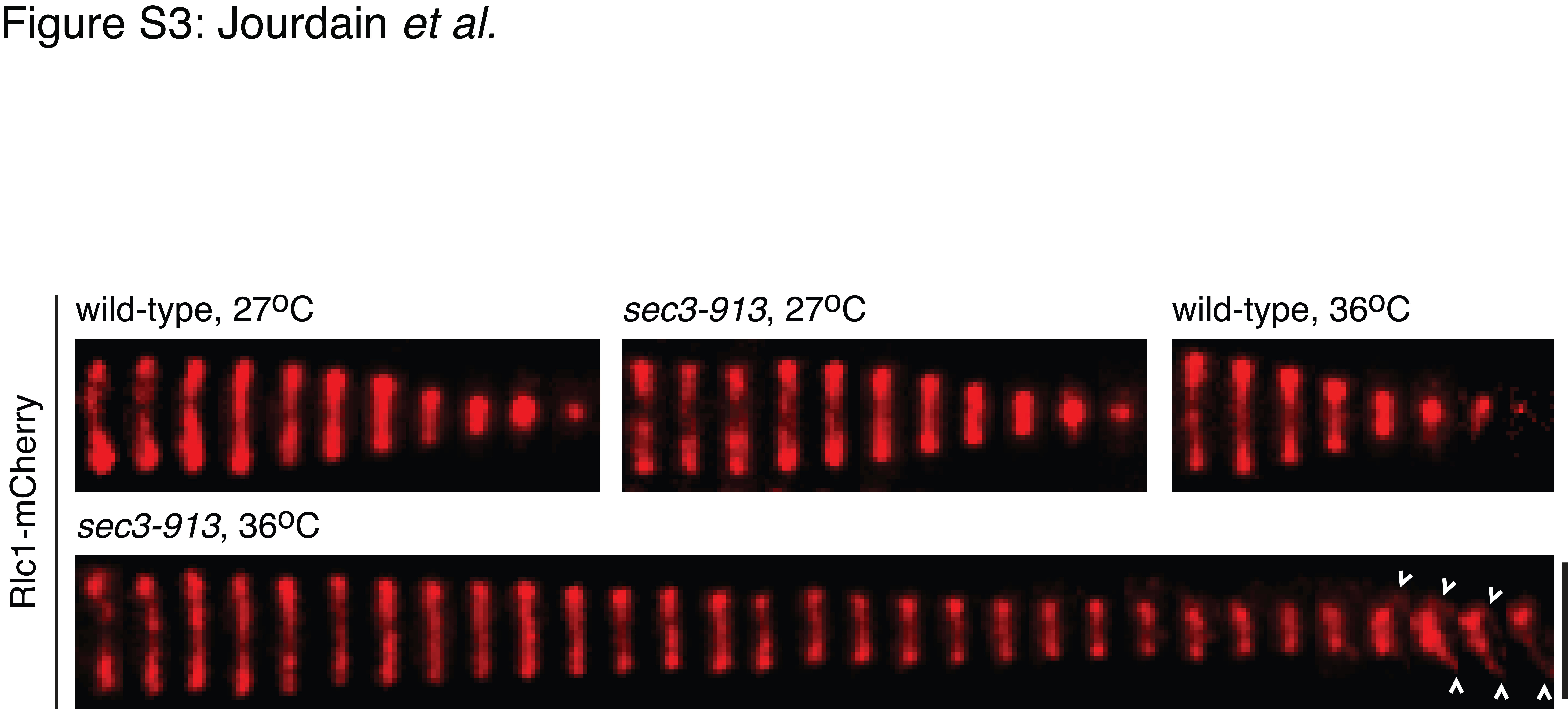


**Figure S3: *Sp*Sec3 controls CAR constriction**

Time-lapse imaging of the CAR marker Rlc1-mCherry observed in representative wild-type and *sec3-913* cells, at 27^o^C or 36^o^C. Four minutes time increments of rings are shown. Arrowheads point at projections of CAR filaments into the cytoplasm, also shown in Figure 5E. Bar = 5 μm.
