# Supplementary material for: Fission Yeast Sec3 Bridges the Exocyst Complex to the Actin Cytoskeleton
Source: Traffic. 2012 Sep 7;13(11):1481–95. doi: 10.1111/j.1600-0854.2012.01408.x (PMC3531892; doi:10.1111/j.1600-0854.2012.01408.x)
Supplement: Supplementary file 6 [file tra0013-1481-SD4.doc]

**Figure S4: Unlike *sec8-1*, *sec3* ts mutants have weak actin cables and delocalized actin patches**

Bodipy-Phallacidin staining of fixed wild-type, *sec3-913,* *sec3-916* and *sec8-1* cells grown at 27^o^C or at 36^o^C for 3.5 h or 6 h. See also Figure 5F. Bar = 5 μm.
